# RNA-Seq of human whole blood: Evaluation of globin RNA depletion on Ribo-Zero library method

**DOI:** 10.1038/s41598-020-62801-6

**Published:** 2020-04-14

**Authors:** Christina A. Harrington, Suzanne S. Fei, Jessica Minnier, Lucia Carbone, Robert Searles, Brett A. Davis, Kimberly Ogle, Stephen R. Planck, James T. Rosenbaum, Dongseok Choi

**Affiliations:** 1 Integrated Genomics Laboratory, Oregon Health & Science University, Portland, Oregon USA; 2 Department of Molecular & Medical Genetics, Oregon Health & Science University, Portland, Oregon USA; 3 Bioinformatics & Biostatistics Core, Oregon National Primate Research Center, Oregon Health & Science University, Beaverton, Oregon USA; 4 OHSU-PSU School of Public Health, Oregon Health & Science University, Portland, Oregon USA; 5 Knight Cardiovascular Institute, Oregon Health & Science University Portland, Oregon, USA; 6 3181 Sam Jackson Park Rd, Oregon Health & Science University, Portland, Oregon United States; 7 Casey Eye Institute, Oregon Health & Science University, Portland, Oregon USA; 8 Department of Medicine, Oregon Health & Science University, Portland, Oregon USA; 9 Legacy Devers Eye Institute, Legacy Health System, Portland, Oregon USA; 10 Graduate School of Dentistry, Kyung Hee University, Seoul, Korea

**Keywords:** Gene expression analysis, Transcriptomics, Biomarkers

## Abstract

Peripheral blood is a highly accessible biofluid providing a rich source of information about human physiology and health status. However, for studies of the blood transcriptome with RNA sequencing (RNA-Seq) techniques, high levels of hemoglobin mRNAs (hgbRNA) present in blood can occupy valuable sequencing space, impacting detection and quantification of non-hgbRNAs. In this study, we evaluated two methods for preparing ribosomal RNA (rRNA)-depleted sequencing libraries for RNA-Seq of whole blood, one of which is also designed to deplete hgbRNAs. Two experiments were performed: one evaluating library performance across 6 human blood samples and the other examining library reproducibility and performance in a two-subject subset. We find that addition of hgbRNA depletion to the rRNA-depletion protocol for library preparation from blood RNA effectively reduces highly abundant hgbRNA reads; however, it does not result in a statistically significant increase in differentially expressed genes in our patient-control study. Bioinformatic removal of globin gene counts in non-hgbRNA depleted libraries provides improvement in overall performance of these libraries. We conclude that use of a standard ribosomal RNA depletion method for library preparation coupled with bioinformatic removal of globin gene counts is sufficient for reproducible and sensitive measurement of both coding and noncoding RNAs in the blood transcriptome.

## Introduction

Blood is a relatively easy to access biofluid that contains information on the physiology and health status of the individual. As a primary conduit of nutrients, cells, small vesicles and molecular signals throughout the body, blood can serve as a reporter for both systemic disease and localized pathological activity associated with many different tissues. Analysis of the blood transcriptome has potential to identify gene expression signatures and other biomarkers for diagnosis, prognosis, and monitoring of treatment response, and to improve understanding of disease mechanisms. Because peripheral whole blood collection is a standardized and routine procedure, the use of whole blood samples in multicenter gene expression studies is more likely to produce reproducible results than the use of time consuming and potentially variable procedures to isolate specific cell populations following blood collection. In addition, whole blood collection and storage products such as PAXgene or Tempus Blood Tubes limit RNA degradation and minimize the risk of cell activation that can occur during a blood cell fractionation step post-collection. However, RNA extracted from whole blood samples contains high levels of hemoglobin RNAs (hgbRNA)^1,2^ from the dominant red blood cell component. These high abundance transcripts can interfere with sensitive measurement of the rest of the blood transcriptome^3,4^.

Studies utilizing microarrays for whole blood expression profiling have demonstrated that sample preparation methods that reduce or block hgbRNAs increase sensitivity of gene detection^5–7^. More recently, it has been reported that globin RNA depletion from blood RNA prior to preparation of polyA+ RNA sequencing libraries results in the detection of thousands of additional transcripts with RNA-seq^2,8^. While use of polyA+ RNA libraries allows comprehensive coverage of polyA+ protein-coding RNAs, these libraries do not provide information on polyA− mRNAs or many noncoding RNAs. For our studies of the blood transcriptome we wished to identify an efficient and reproducible library preparation method that would support measurement of a broad range of blood RNAs, including noncoding RNAs and low abundance transcripts.

Sequencing library methods have evolved to effectively remove highly abundant ribosomal RNA (rRNA) species through pre-selection of poly A+RNAs or capture and removal of rRNA during library preparation^9^. Routine implementations of these methods, however, do not remove highly abundant protein-coding RNAs such as the hemoglobin gene transcripts present in whole blood. A library preparation method that simultaneously removes both hgbRNA transcripts and rRNAs, Ribo-Zero Globin (Illumina) is available. Ribo-Zero Globin (originally distributed as the Globin-Zero Gold rRNA Removal kit) is a modification of the Ribo-Zero Gold rRNA removal protocol which incorporates a panel of human hgbRNA oligos along with nuclear and mitochondrial rRNA oligos to allow simultaneous removal of both hgbRNA transcripts and rRNAs. These oligos are hybridized with total RNA followed by magnetic bead-based removal of the resulting complexes prior to RNA fragmentation and cDNA synthesis for sequencing library preparation. In the present study we wished to determine if the Ribo-Zero Globin (Globin-Zero) library method provided an effective approach to RNA-Seq of whole blood that would allow measurement of protein-coding and non-coding RNAs in a reproducible and sensitive manner. We hypothesized that removal of hgbRNAs along with rRNAs for RNA-Seq of blood would result in detection of more genes and less variation among samples due to large differences in hgbRNA levels. Therefore, we compared Globin-Zero to the standard Ribo-Zero Gold method to determine if Globin-Zero effectively removed hgbRNA transcripts and produced reproducible libraries that allow detection of additional genes.

We designed a small exemplar study utilizing blood samples collected from an age and diagnostically mixed donor group to evaluate library method effectiveness in revealing gene expression differences among biological replicates. We included samples from healthy controls and patients with sarcoidosis, an inflammatory disease in which we have previously demonstrated characteristic gene expression profiles in blood with potential for molecular diagnosis^10^. We examined which hgbRNAs were removed by the Globin-Zero method, effects of reducing RNA input to 250 ng from 900 ng, and the impact of library method on gene detection and differentially expressed genes (DEG). In a separate experiment, we further examined RNA-Seq reproducibility with the different methods by preparing technical replicate libraries from blood RNA of one male and one female subject. We also evaluated differences in detection sensitivity with and without bioinformatic removal of hemoglobin gene counts from the RNA-Seq data. The results of our study confirm that the Globin-Zero method is very effective at reducing hgbRNA transcripts in human blood samples. However, we were unable to demonstrate an improvement in DE gene measurement with the Globin-Zero library method compared to Ribo-Zero Gold in our multi-sample gene expression study.

## Results

### Experimental design and analysis strategy

**Figure 1:**
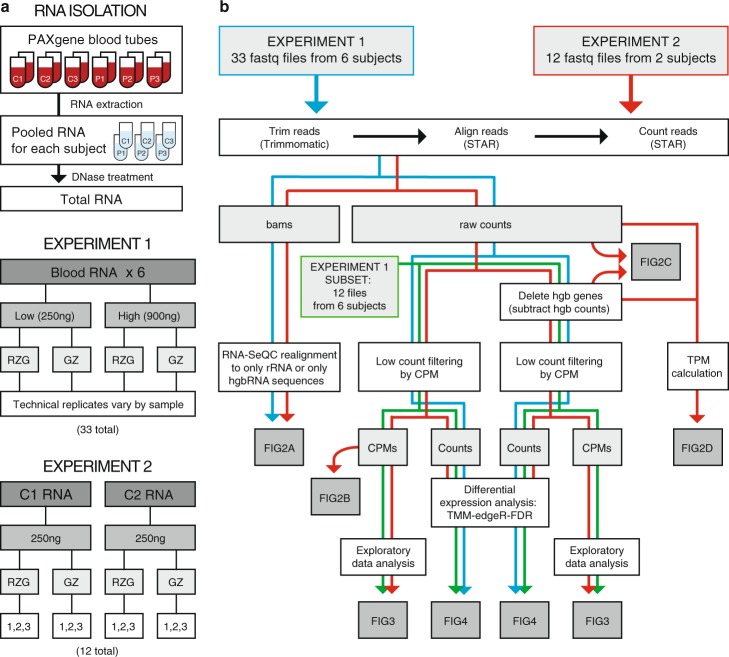
Experimental Design and Analysis. (**a**) Blood RNA preparation steps from 6 human subjects: 3 controls (**c**) and 3 patients (P). RNA was used for preparing libraries with Ribo-Zero Gold (RZG) or Globin-Zero (GZ) kits as shown. In Experiment 1, a total of 33 libraries were prepared with either 250 ng total RNA for low (L) input or 900 ng for high (H) input. In Experiment 2 technical replicate libraries were prepared using 250 ng RNA from C1 and C2 with either RZG or Globin-Zero for a total of 12 libraries. Individual RNA-seq data files generated from the libraries shown in B and C were named according to the following convention: sample ID_RNA input_library method_replicate number, e.g., C1_L_GZ_1. (**b**) Fastq files were assessed for quality metrics, aligned and analyzed as shown. Correspondence between data analysis steps and results shown in Figs. 2–4 is indicated.

Blood was collected in PAXGene Blood RNA Tubes from three sarcoid patients (P) and three healthy volunteers (C). Total RNA isolated from two PAXGene tubes per donor was pooled followed by a second DNase treatment (Fig. 1a). RNA extractions and DNase-treatments were each performed in a single batch, thus limiting potential batch effects among samples during blood RNA isolation. RNA quality was good with Bioanalyzer integrity values ranging from RIN of 7.7 to 8.8.

We tested two library preparation methods for the whole blood RNA samples: TruSeq Stranded Total RNA Library Prep Kit with Ribo-Zero Gold and the TruSeq Stranded Total RNA Library Prep Kit with Ribo-Zero Globin. In this report, we abbreviate the RNA depletion and library preparation protocols used as RZG (Ribo-Zero Gold) and Globin-Zero (Ribo-Zero Globin) and use the term ‘library method’ to refer to all steps in library generation from RNA depletion to PCR-amplified DNA library. The methods evaluation study was divided into two experiments. Experiment 1 examined the impact of library method and RNA input on RNA-Seq performance across six independent blood samples (3 patients and 3 healthy controls) using a balanced block design (½-fractional factorial design^11^) for sequencing (Fig. 1a and Supplementary Fig. S1). RNA input amounts of 900 ng (High) and 250 ng (Low) were tested. Due to limiting amounts of RNA for two of the patient samples, three high input RZG libraries could not be generated resulting in 33 libraries for RNA-Seq instead of 36.

Experiment 2 was set up to evaluate technical reproducibility of the two library methods and evaluate the impact of method on measured differences between the two biological samples when tested in replicate. Globin-Zero and RZG libraries were prepared from each of two blood samples (healthy subjects C1 and C2) in triplicate and sequenced in a block design (Fig. 1a and Supplementary Fig. S1).

Sequence data from both experiments were aligned, quality-checked and analyzed as outlined in Fig. 1b. Data visualization and differential expression (DE) analysis were performed with and without the removal of hemoglobin gene counts from the gene count data. This was done to assess the impact of high levels of hemoglobin gene counts in RZG libraries on between-method data distribution patterns and data normalization for DE measurements. A paired analysis of RNA-seq data generated with either Globin-Zero or RZG from each of 6 human donors was used to measure same sample differences in relative gene levels as a function of library method in a selected subset of libraries from Experiment 1. To examine the impact of library method on DE analysis of our patient and control samples (Experiment 1), we examined interaction of library method, disease status and RNA input while accounting for potential flow cell and lane effects. Finally, we used our technical replicate experiment (Experiment 2) to examine reproducibility with each library method and how gene expression measurements between C1 and C2 samples were affected by method.

### RNA-Seq data quality and impact of globin RNA depletion

**Figure 2:**
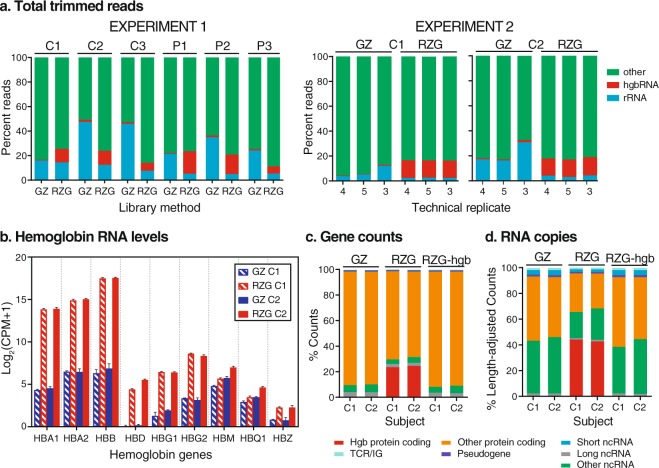
Read distributions across methods and samples. (**a**) Estimated fraction of reads that map to hemoglobin (Hgb) genes, rRNA, and Other in method-paired datasets from 6 human donors (Experiment 1) and technical replicate datasets from C1 and C2 donors (Experiment 2) with either Globin-Zero (GZ) or Ribo-Zero-Gold (RZG). Proportions were estimated by mapping a subset of total trimmed reads to only Hgb gene sequences or only rRNA gene sequences, intentionally including multi-mapped reads. (**b**) HgbRNA levels in C1 and C2 libraries. Counts per million (CPM) expression levels measured in Experiment 2 GZ and RZG replicate libraries for Hgb genes were used to calculate the average log2 CPM for each gene in the C1 and C2 samples with each library method. Hgb gene family members with fewer than 20 raw counts in all libraries are not shown. (**c**) Ensembl gene biotype (https://uswest.ensembl.org/info/genome/genebuild/biotypeshtml.) proportions from Experiment 2 utilizing the raw gene counts (number of uniquely-mapped reads that unambiguously map to only one gene) that were used for subsequent quantitative analysis. Technical replicates are averaged. RZG-Hg indicates gene counts from RZG libraries after bioinformatic removal of Hgb gene counts. (**d**) Gene biotype proportions of counts after TPM calculation which estimates the number of RNA transcripts from each gene by not only normalizing the raw gene counts by library depth as CPM does but also by accounting for gene length; this permits count comparison of differently-sized transcripts within a sample. Technical replicates are averaged.

To determine the effect of hgbRNA depletion on RNA-Seq data quality and compare performance between Globin-Zero and RZG library preparation methods, sequence data metrics were assessed before and after read alignment to the human genome assembly. Quality checkpoints were monitored in accordance with the recommendations of Conesa *et al*.^12^, utilizing both FastQC^13^ and RNA-SeQC^14^. Mapping rates of total reads to both rRNA and hgbRNA were estimated by using the rRNA estimation functionality in RNA-SeQC which allowed the inclusion of both uniquely mapped and multi-mapped reads for these two gene families (Fig. 2a). The rRNA sequences provided with the RNA-SeQC tool and hgbRNA sequences from Ensembl (r91) were utilized, specifically ten protein-coding hemoglobin subunit genes and two pseudogenes (Supplementary Table S1). Total reads for Experiment 1 libraries ranged from 56 million to 72 million reads (Supplementary Table S2). FastQC revealed high duplicate read rates in two of the Globin-Zero libraries in Experiment 1 and RNA-SeQC metrics generated on the aligned data showed that these two libraries, C1_L_GZ_1 (C1_Low Input_Globin-Zero_rep1) and C1_H_GZ_1 (C1_High Input_Globin-Zero_rep1) had reduced gene coverage compared with the other Experiment 1 libraries and a very high percentage of rRNA reads (79 and 68%, respectively). They also had high levels of hgbRNA reads compared with the other Globin-Zero libraries (Supplementary Table S2) suggesting that both rRNA and hgbRNA removal had been inefficient. The same libraries appeared as outliers using multidimensional scaling (MDS)^15^ to visualize library similarities (Supplementary Fig. S2). Given these observations, data from C1_L_GZ_1 and C1_H_GZ_1 were excluded from further analyses. MDS visualization of the remaining 31 libraries shows a less skewed distribution among libraries with close clustering of technical replicates.

In Experiment 1, Globin-Zero libraries had an average of 1.1% of reads mapping to hgbRNAs and RZG libraries 12.3%. In the non-hgbRNA depleted libraries, hgbRNA reads from individual RNA samples ranged from 5.9% to 18.5% of total reads (Supplementary Table S2). Surprisingly, we also observed differences in library rRNA content between the two methods with Globin-Zero libraries having a mean rRNA mapping rate of 28.3% and RZG libraries 7.2%. The percentage of total RNA-Seq reads aligning to hgbRNA and rRNA is illustrated in Fig. 2a for pairs of RNA input-matched Globin-Zero and RZG libraries prepared from each of 6 human blood samples. Following gene quantification which discards intergenic and multi-mapped reads, the percent of raw gene counts in Experiment 1 libraries aligning to hemoglobin genes ranged from 10 to 33% in RZG libraries and was reduced to less than 0.1% with Globin-Zero (Supplementary Table S2).

RNA-Seq data quality observed in Experiment 2 libraries (Table 1 and Supplementary Table S2) was similar to that seen with Experiment 1. The technical replicate design included in Experiment 2 allowed us to confirm a performance trend observed with Experiment 1: libraries prepared with the Globin-Zero method had increased rRNA reads compared with RZG libraries (3.9% to 30.9% in Globin-Zero libraries, 2.4% to 4.3% in RZG). In addition, the fraction of rRNA reads measured in the Globin-Zero libraries was more variable than that seen with RZG (Fig. 2a and Table 1). The higher rRNA rates observed in Globin-Zero libraries contribute to the higher intergenic rates seen in both Experiment 1 and Experiment 2 Globin-Zero libraries since many rRNAs are not annotated and their reads are therefore classified as intergenic. In Experiment 2, for example, exonic rates in Globin-Zero libraries averaged 45% for C1 and 39% for C2 compared to RZG averages of 53% for both; intergenic rates were increased in Globin-Zero (11% and 22%) compared to RZG (6% and 8%). The average proportion of intergenic reads with the Globin-Zero protocol in our study is similar to that reported by Zhao *et al*.^16^ in their analysis of a single pooled human blood sample prepared with the Globin-Zero library method.

**Table 1:** Experiment 2 RNA-Seq performance metrics reveal differences between library methods in rRNA and hemoglobin mRNA levels.

			Alignment Metrics							Gene Counts	
Blood Sample	Library Method	Technical Replicate	Total reads	rRNA rate^a^	Globin rate^a^	Total mapped	Duplicate rate^b^	Exonic rate^c^	Intergenic rate^c^	Total counts^d^	Globin Rate^e^
C1	Ribo-Zero Globin	1	75,889,973	**3.91%**	0.46%	74,970,455	56.86%	46.49%	8.53%	26,940,402	0.03%
		2	68,187,212	**5.17%**	0.47%	67,382,218	57.07%	45.93%	9.61%	23,852,287	0.02%
		3	66,912,681	**12.16%**	0.79%	65,920,965	59.11%	43.00%	14.99%	21,312,218	0.02%
	Ribo-Zero Gold	1	62,258,328	2.38%	**14.17%**	61,590,653	58.87%	53.62%	6.02%	23,277,919	**23.70%**
		2	63,705,502	2.52%	**14.04%**	63,053,394	60.15%	52.83%	6.36%	23,427,822	**23.31%**
		3	64,213,439	2.42%	**13.99%**	63,462,386	60.39%	52.82%	6.44%	23,582,022	**23.29%**
C2	Ribo-Zero Globin	1	61,117,132	**17.19%**	0.88%	59,981,781	60.00%	40.97%	18.55%	17,569,235	0.03%
		2	66,744,713	**16.27%**	0.91%	65,499,404	62.22%	41.02%	17.84%	19,226,041	0.02%
		3	62,514,672	**30.89%**	1.65%	60,682,926	71.18%	34.45%	28.66%	13,765,481	0.03%
	Ribo-Zero Gold	1	64,728,692	4.01%	**13.90%**	63,842,383	60.71%	52.01%	7.69%	22,556,548	**23.87%**
		2	67,886,975	2.97%	**14.08%**	67,119,332	60.32%	52.75%	6.86%	24,114,916	**23.80%**
		3	66,636,958	4.33%	**14.66%**	65,293,327	62.61%	53.46%	8.14%	22,931,860	**25.83%**

^a^Estimated % of total reads via independent alignment of reads to only rRNA and hemoglobin sequences; ^b^% of mapped reads; ^c^% of mapped non-duplicate reads^14^; ^d^uniquely mapped reads unambiguously assigned to one gene; ^e^% of gene counts.

Rates in bold text highlight elevated rRNA detection in the Ribo-Zero Globin (Globin-Zero) libraries and the high hemoglobin mRNA levels in the Ribo-Zero Gold (RZG) libraries.

Following read alignment and gene quantification with STAR^17^, we examined the gene identities of hemoglobin transcripts removed with the Globin-Zero kit. Counts per million (CPM) expression levels of hemoglobin genes in Experiment 2 libraries were determined and average log_2_ (CPM + 1) are shown in Fig. 2b (hemoglobin gene family members with fewer than 20 reads in all Experiment 1 and Experiment 2 libraries are not shown and are considered unexpressed). As expected, the most abundant hgbRNAs in the blood samples correspond to the α- and β-hemoglobin genes. The Globin-Zero library preparation resulted in > 1,000-fold decrease in CPM for these three hemoglobin genes. Reductions in reads mapping to the other hemoglobin genes were also seen. A similar pattern of reduction was observed for the Experiment 1 libraries (Supplementary Table S2).

### RNA biotype distributions with Globin-Zero and Ribo-Zero Gold library methods

Gene and transcript annotations for aligned counts were grouped according to Ensembl^18^ biotype categories. Biotype categorization of raw gene counts (number of uniquely mapped reads unambiguously assigned to one gene, averaged across triplicates) for the Experiment 2 C1 and C2 libraries showed that functional distributions were similar for the two library methods (Fig. 2c, Supplementary Table S3) with the vast majority of gene counts corresponding to protein-coding genes and 6 to 10% to non-coding RNAs (ncRNA). Proportionally, Globin-Zero libraries contain more non-hemoglobin protein-coding and ncRNA gene counts. Following bioinformatic removal of hemoglobin gene counts, however, the biotype pattern in RZG libraries is very similar to Globin-Zero. Adjusting the raw gene counts to account for gene length permitted an estimation of the proportion of transcripts belonging to each biotype category. Utilization of the Transcripts Per Million (TPM) calculation showed that in C1 and C2 RZG libraries, over 40% of quantified transcripts belong to the relatively short hemoglobin genes, 30% belong to other protein-coding genes, and the rest primarily belong to ncRNA (Fig. 2d, Supplementary Table S3). Conversely, in Globin-Zero libraries, half of the quantified transcripts belong to protein-coding genes while most of the rest belong to ncRNA. Again, bioinformatic removal of hemoglobin gene counts in RZG libraries produces a transcript biotype pattern similar to Globin-Zero.

### Concordance and reproducibility of gene expression with Globin-Zero and Ribo-Zero Gold library methods

**Figure 3:**
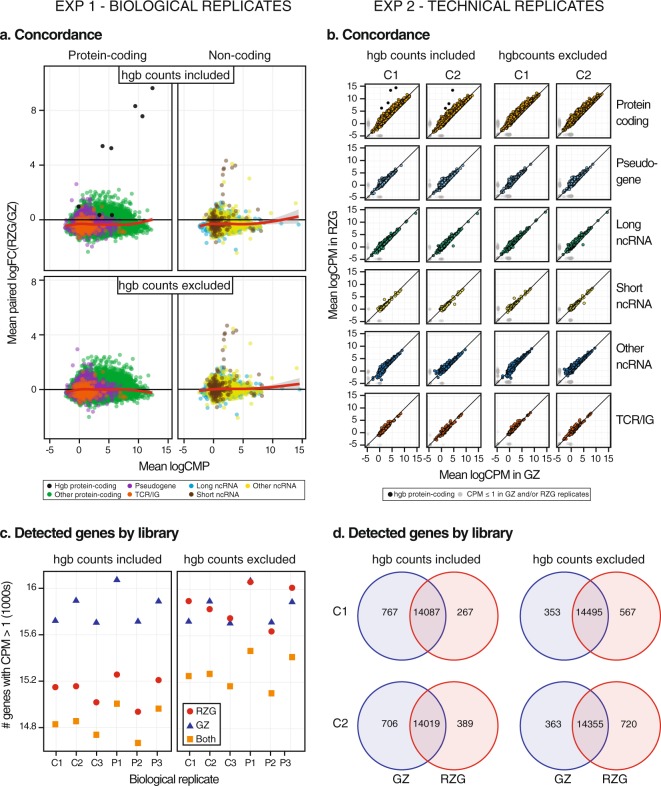
Library method affects within-sample concordance and gene detection. (**a**) MA plots averaged over 6 Experiment 1 library method pairs. Each library pair is matched for RNA input amount. Mean paired log FC is averaged over 6 pairs and mean log CPM of protein-coding genes is averaged over all 12 libraries. (**b**) Correlation plots of gene expression (averaged log2CPM) for Experiment 2 C1 and C2 technical replicates prepared with either GZ or RZG library method. (**c**) Genes detected at CPM > 1 in a single pair of RZG and GZ libraries prepared from each of the 6 samples in this study. (**d**) Genes detected at CPM > 1 in 3 out of 3 library replicates for either C1 or C2 using libraries prepared in Experiment 2. Venn diagrams show genes detected in common with GZ and RZG or uniquely with one library method. Detection analysis was done with all mapped gene counts included or after removal of all hemoglobin gene counts.

To visualize differences between RZG and Globin-Zero measurements in Experiment 1 we selected a pair of input-matched libraries for each sample (6 method pairs in total) and generated MA plots with and without bioinformatic removal of hemoglobin gene counts. MA plots between methods were similar across the 6 samples so the averaged paired logFC (RZG/GZ) by average logCPM is shown (Fig. 3a). The loess curve (red line) is centered below 0 when hemoglobin gene counts are retained and moves up to 0 once hemoglobin gene counts are removed. Interestingly, we observe a pattern that indicates a reduced CPM measurement in the Globin-Zero libraries for a subset of genes. Since the loess line is close to 0 across all levels of abundance despite this subset, it appears the majority of genes fall symmetrically around the horizontal 0 line and there is not a systematic difference in CPM between the two library methods after removing hemoglobin gene counts. Therefore, bioinformatic removal of hemoglobin gene counts removes the hemoglobin gene outliers in Fig. 3a and adjusts CPM calculations with RZG to be closer to Globin-Zero CPMs on average, but it does not correct the observed pattern of reduced CPM measurements for a subset of genes in Globin-Zero libraries compared to RZG libraries.

We examined how gene expression measurements correlated between library types in Experiment 2 using between-method scatter plots of averaged log_2_ CPM values from three technical replicates with each method for samples C1 and C2 (Fig. 3b). While overall concordance is high (Pearson correlation r ≥ 0.97) and similar across all biotype categories, the plots show an asymmetric pattern in which a subset of genes fall slightly above the diagonal (higher CPM in RZG libraries). This asymmetric pattern is most pronounced in protein-coding genes across all abundance levels and is seen with and without bioinformatic hemoglobin gene count removal. This indicates a reduced CPM measurement in the Globin-Zero libraries for a subset of genes in addition to the expected large reductions in hemoglobin gene counts similar to what was seen with the Experiment 1 data set.

In order to assess technical reproducibility with the different library preparation methods, within-method variability across the technical replicates was assessed by measuring the coefficient of variation (CV) in raw gene counts per gene. A small, but significant, increase in variation with Globin-Zero library preparation (p < 0.001 by the Wilcoxon signed-rank test) was observed. The median CV across technical replicates for RZG libraries was 0.24 for C1 and 0.22 for C2, and across Globin-Zero libraries, 0.26 and 0.33, respectively.

### Difference in gene detection sensitivity with RNA input

The impact of RNA input amount on transcript detection sensitivity in Experiment 1 was examined by counting the number of genes above a fixed CPM threshold in each library. Genes with consistently low counts across all libraries are usually removed for downstream analysis since they are unlikely to be biologically important or to be assessed as significantly DE. Chen *et al*.^19^ recommends filtering genes that do not have at least 10–15 counts or equivalent CPM values. We selected a filter of >1 CPM (corresponding to approximately 15 to 25 raw counts in our dataset) as our threshold for detection. Genes are considered uniquely detected if present at >1 CPM in all libraries prepared with one of the input amounts and measured at ≤1 CPM in at least one of the alternative input libraries. In a combined analysis of all Experiment 1 control libraries (Globin-Zero and RZG), 0.3% more genes (46) were detected with 900 ng RNA input compared to 250 ng out of approximately 14,000 genes detected. While this result suggests a slight increase in gene detection with 900 ng RNA, the actual increase in gene number is very small (this conclusion is further supported by the results of the multifactor analysis of Experiment 1). Therefore, we elected to prepare libraries with 250 ng RNA input in Experiment 2. We reasoned that the further evaluation of libraries prepared with 250 ng RNA would provide useful information for whole blood transcriptome studies in which RNA is limited.

### Difference in gene detection with RNA-depletion protocol used for library preparation

Selected library method pairs (one Globin-Zero and one RZG library) for each of the 6 samples in Experiment 1 were used to evaluate gene detection using detection criteria of >1 CPM in at least one of the libraries. Figure 3c shows gene detection for the individual sample-library pairs. For each sample, approximately 15,000 genes are detected in common with the two methods. The overall detection rate is greater with Globin-Zero by about 5% to 6% (774 to 931 additional genes). The benefit of Globin-Zero on detection efficiency is largely lost when hemoglobin gene counts are removed prior to data transformation to CPM.

Using Experiment 2 data we determined reproducible gene detection with each library method. In total, 36,395 genes were counted at least once (CPM > 0) in the Experiment 2 data set. Applying a relatively stringent filter of CPM > 1 in all 3 method replicates for each sample, 14,087 genes were reproducibly detected in C1 with both library preparations and 14,019 genes in C2 (Fig. 3d). Globin-Zero libraries allowed reproducible detection of an additional 767 genes in C1 and 706 genes in C2, while only 267 and 389 genes were uniquely detected at CPM > 1in RZG libraries (Supplementary Table S4). This corresponds to approximately 3% more genes detected with the Globin-Zero method. However, after bioinformatic removal of counts corresponding to hemoglobin genes from all datasets, there was an increase in genes detected in common with the two library methods, 14,495 genes in C1 and 14,355 genes in C2, and a greater number of genes uniquely detected at CPM > 1 in the RZG libraries, 214 more genes detected in C1 and 457 in C2. We binned the uniquely detected genes according to abundance level in RZG (high: 3 ≤ mean (logcpm in RZG); medium: −1 ≤  mean(logcpm in RZG) < 3; and low: mean(logcpm in RZG) < −1 or not measured in RZG). In the absence of hemoglobin gene count removal, 22% of genes uniquely detected with Globin-Zero correspond to low abundance transcripts and 78% to medium abundance (Supplementary Fig. S3). The majority of genes detected only in Globin-Zero after bioinformatic removal of hemoglobin gene counts were of medium abundance, 94% (318 genes). These results suggest that, with bioinformatic removal of hemoglobin gene counts, the advantage of Globin-Zero in detecting additional low abundance genes is small.

### Impact of library method on measured gene expression levels

**Figure 4:**
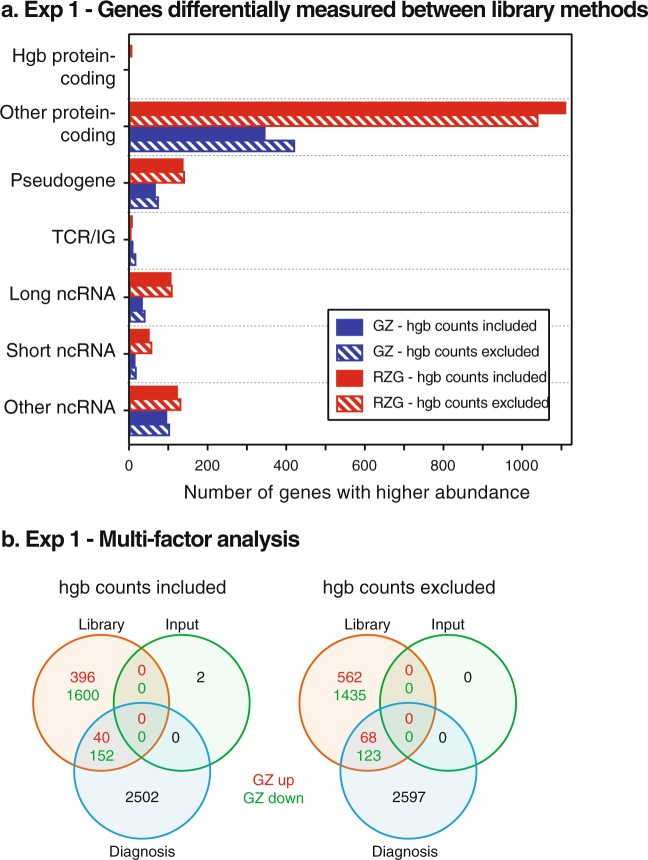
Impact of library method on differentially expressed gene measurements. Differentially-expressed genes (DEG) were measured as described with RNA-Seq data from Experiment 1 libraries. (**a**) Biotypes of genes measured as DE between library methods in a paired analysis of 6 blood samples. (**b**) DEG from comprehensive, multi-factor analysis of the Experiment 1 libraries (patients and healthy controls). Green numbers library circle indicates number of genes up in Globin-Zero relative to RZG and purple down in Globin-Zero. No significant genes were detected by the interactions between disease diagnosis and library method in Experiment 1.

We further examined how library method affected gene measurements by performing a paired analysis (RZG × Globin-Zero) of the 6 sample method pairs from Experiment 1. We found 2,138 genes measured at significantly different levels between methods (Supplementary Table S5) with more genes present at higher abundance in the RZG libraries. The min log2FC – max log2FC for RZG/GZ is −4.404 to 10.022 with highest FC for the hemoglobin genes as expected. Figure 4a shows the number of genes in the different biotype categories that were increased in either Globin-Zero or RZG libraries. More than twice as many protein-coding genes were present at significantly higher levels in RZG libraries compared to the number of genes increased in Globin-Zero. Removal of hemoglobin gene counts prior to CPM calculation and analysis has only a very minor impact on this imbalance with a total of 2,197 genes measured as significantly DE.

### Impact of library method on differential expression among blood donor samples

Using Experiment 1 data, we examined the effects of library method, disease status, RNA input, and interactions of library method and disease status while accounting for potential flow cell and lane effects. We would expect to see an interaction effect which is a synergistic or antagonistic effect on top of the additive main effects, if the library method has a significant impact on the detection of DEG. The small size of our study limits the ability to detect disease-related differences between the patient and control groups, but we were able to use the data we had from 6 independent blood donors comprising two subject groups (differing in both diagnosis and age) to explore the impact of RNA input and library method on measurable differences between the groups. Genes were considered unexpressed if measured at ≤1 CPM in all 31 libraries and removed prior to DE analysis. Significant DE was defined as the false discovery rate (FDR) adjusted p-value < 0.05 and fold change (FC) > 1.5.

There were 2,694 DEG (1,499 up- and 1,195 down-regulated genes) in the patient group (Fig. 4b), however, our analysis did not demonstrate any gain in statistical power in testing patient versus control groups by switching from RZG to Globin-Zero. There were no genes with significant interactions between disease status and library method. This indicates that the Globin-Zero library method could not reveal any extra genes for the patient-control comparison. Bioinformatic removal of hemoglobin gene counts prior to DEG did not alter this outcome. We did, however, observe a substantial library effect on gene expression measurements with 2,188 (12.0%) significant DEG between library methods; 80% of those genes were decreased in Globin-Zero whereas we had expected that only hemoglobin genes would be affected.

In a further use of our technical replicate study to explore library-associated differences in gene expression measurements between the C1 and C2 samples, we performed DE analyses of the replicates with each library method. In independent difference assessments, 2,005 and 2,320 DEG were found between C1 and C2 with RZG and Globin-Zero, respectively (Supplementary Fig. S4 and Supplementary Table S6); of these genes 1,426 were identified with both library methods. After hemoglobin gene count removal DEG with RZG increased, but Globin-Zero still identified 8.4% more genes than RZG.

## Discussion

Peripheral blood provides a rich source of RNA transcripts reflective of cell diversity and function. However, the blood cell population is dominated by red blood cells that are not of primary interest for many studies and contribute high levels of hgbRNA to the blood transcriptome. These abundant hgbRNAs can compete for sequencing space in RNA-Seq experiments and may negatively affect detection of lowly expressed genes. Therefore, methods that reduce hgbRNAs in the blood sample have been sought. One approach is to remove red blood cells from whole blood samples prior to RNA extraction; however, methods which fractionate blood cells prior to RNA isolation require additional processing time and handling expertise and can lead to post-collection changes in expression^3,20^. For multi-center studies, blood collection and preservation approaches that ensure RNA is stabilized at the time of blood collection in the clinic have clear advantages^21,22^. Therefore, methods which reduce the amount of hgbRNA transcripts in RNA extracted directly from whole blood for RNA-Seq studies have been explored^7,8,16^. Studies in which blood RNA was treated with GLOBINclear to remove hgbRNAs followed by polyA+ library preparation have demonstrated increases in detection of low abundance gene transcripts^2,7,8^. However, this approach limits transcriptome profiling to polyA+ RNAs. Library preparation methods that remove highly abundant RNAs, such as rRNA, but preserve the complex RNA transcriptome, maintaining both polyA+ and polyA− mRNAs and regulatory noncoding RNAs, expand the scope of gene expression studies and are widely used. The Globin-Zero library kit offers the ability to remove both rRNA and hgbRNA as the first step in library preparation for RNA-Seq.

In our RNA-Seq study of blood samples from 6 different individuals, use of a standard rRNA removal kit and Tru-Seq stranded library preparation (RZG) produced high quality libraries. However, up to one-fifth of all sequence reads corresponded to hgbRNAs. Furthermore, up to 33% of uniquely mapped gene counts in RZG libraries mapped to the hemoglobin genes, and hgbRNA levels varied up to 3-fold among subjects. We found that Globin-Zero was highly effective at removing hgbRNA transcripts from RNA-Seq libraries. This resulted in an increase in unique gene detection based on a commonly used gene count threshold (>1CPM), but fewer additional genes than anticipated. In addition, when all hemoglobin gene counts were bioinformatically removed from the Globin-Zero and RZG data, the detection rate advantage seen with the Globin-Zero method was lost. In another methods comparison study working with whole blood samples, sequencing libraries were prepared using polyA+ RNA with and without the prior removal of hgbRNA with GLOBINclear. The authors report that several thousand additional genes were detected with GLOBINclear removal of hgbRNA compared to non-depleted RNA in a pair of libraries prepared from a single sample^8^. Our detection comparison of RZG and Globin-Zero libraries for 6 sample pairs showed that in the absence of hemoglobin gene count removal about 800 to a thousand additional genes were detected in Globin-Zero libraries. This suggests that the increase in gene detection with the Globin-Zero protocol compared to non-hgbRNA-depleted RZG libraries is less than that seen with the combination of GLOBINclear removal of hgbRNAs followed by library construction with polyA+ selected RNA.

We find a large number of genes, predominately protein-coding, are present at reduced levels in Globin-Zero libraries relative to RZG (approximately 2,000 significantly DE). This suggests that the addition of an hgbRNA removal step with Globin-Zero results in the reduction of reads for some non-hgbRNAs as well. A broad reduction in counts for genes other than the hemoglobin genes was also reported by Shin *et al*.^8^ in their study using GLOBINclear to remove hgbRNA transcripts prior to polyA+ selection and library preparation. We suspect that these reductions in other transcripts are due to cross-hybridization with the α- and β-globin probes present in the Globin-Zero and GLOBINclear kits.

DE analyses of libraries prepared in Experiment 1 showed that the removal of hgbRNAs with Globin-Zero did not result in an increase in identification of DEG between patients and controls compared to the standard RZG protocol used in this study. While our subject sample number is small, if one library method considerably improved DEG sensitivity in Experiment 1, our design should have detected it. Independent comparisons of blood RNA samples from two individuals in Experiment 2 using method replicates showed only a small increase in differentially-measured genes with the Globin-Zero method (approximately 8%). Our assessment of sequencing library performance characteristics suggests that one of the reasons the Globin-Zero method did not result in a large increase in gene detection and DEG may be a concomitant reduction in efficiency of rRNA removal, resulting in relatively fewer exonic reads with the Globin-Zero protocol compared to RZG. DEG analysis with the Globin-Zero method may be further impacted by variability in the amount of rRNA retained in the GZ libraries. Reduction in the levels of many non-hgbRNAs with Globin-Zero may also contribute to its inability to detect more DE genes.

## Conclusions

Our results confirm that Globin-Zero effectively removes high abundance human hgbRNA transcripts from RNA-Seq libraries. However, increases in rRNA content in Globin-Zero libraries relative to the RZG method and a small increase in technical variation with Globin-Zero protocol are also seen. Nonetheless, Globin-Zero library preparation results in the detection of additional genes compared to RZG libraries when routine data filtering protocols are applied, if hemoglobin gene counts are not removed prior to data normalization. A small increase in DE sensitivity with Globin-Zero is seen in a two-sample technical replication experiment, but this increase in sensitivity with Globin-Zero did not translate to our patient-control study. In addition, we observed an impact of the hgbRNA removal protocol on the measured abundance levels of hundreds of genes in addition to the hemoglobin genes.

Overall, our results indicate that the benefit of the combined hgbRNA and rRNA removal with Globin-Zero on gene expression measurements is small. Alternative approaches that remove hgbRNAs and rRNA in separate steps prior to cDNA synthesis may offer useful options for those seeking to profile the expression of both polyA+ and polyA− genes in whole blood samples, but the two-step depletion/enrichment process adds time and cost, and increases risk of RNA degradation and loss. In our study both the Globin-Zero and RZG methods produced informative and largely overlapping RNA-Seq data for whole blood samples. We find that RZG when combined with bioinformatic removal of hemoglobin gene counts is an effective library preparation method for the analysis of blood RNA.

## Methods

### Samples

Blood collection from patients and healthy controls was performed in accordance with a protocol approved by the Institutional Review Board of Oregon Health & Science University. Blood was collected in PAXgene Blood RNA Tubes (PreAnalytix/Qiagen) and processed according to manufacturer recommendations prior to storage at −80 °C. Blood was collected from three sarcoid patients (P1, P2, and P3; average age 59) and three healthy volunteers (C1, C2, and C3; average age 28). All subjects were female except C2. Two blood tubes were collected per donor.

### RNA isolation and quality assessment

PAXgene tubes were thawed and incubated at room temperature overnight. RNA was extracted according to manufacturer instructions using PAXgene Blood RNA Kits (Qiagen) and a QIACube sample-processing robot (Qiagen). Isolated RNA from the same donor was pooled, and a second DNase treatment was performed using DNase 1 (Zymo Research) according to manufacturer instructions followed by RNA repurification and concentration with RNA Clean & Concentrator kits (Zymo Research). RNA concentrations were initially determined by UV measurement. RNA integrity and concentration for library preparation were determined by analysis on an RNA 6000 NanoChip using a 2100 Bioanalyzer (Agilent Technologies). RIN values were 8.6, 8.3, 8.8 for control (C) samples and 7.7, 8, 8.5 for patient (P) samples.

### Library preparation and RNA-Seq

Libraries were prepared with total RNA according to manufacturer instructions with either the TruSeq Stranded Total RNA Library Prep Kit with Ribo-Zero Gold (Illumina, Cat. No. RS-122-2301) or the TruSeq Stranded Total RNA Library Prep Kit with Ribo-Zero Globin Set A (Illumina, Cat. No. RS-122-2501). Experiment 1 libraries were prepared with either 900 ng or 250 ng of total RNA. All Experiment 2 libraries were prepared with 250 ng of total RNA.

Library quality was confirmed by size analysis on 2200 Tapestation with D1000 ScreenTape. All libraries were within an average size range of 360 to 380 bp. Library concentrations were determined using the KAPA Library Quantification Kit for Illumina platforms (KapaBiosystems) on a StepOne or a StepOnePlus Real Time PCR Workstation (Thermo Fisher) and pooled at 4 samples per lane. Single-end, 100 cycle sequencing was performed on a HiSeq 2500 platform (Illumina). Libraries were pooled according to block designs shown in Supplementary Fig. S1.

### Data processing and QC

Sequence data quality was evaluated using FastQC^13^ combined with MultiQC^23^ (http://multiqc.info/). Sequence files were imported into and processed through our LabKey^24^ and DISCVR-Seq-based system (https://github.com/BimberLab/discvr-seq). Trimmomatic^25^ was used to remove any remaining Illumina adapters. Reads were aligned to the Homo_sapiens.GRCh38.p10 genome assembly in Ensembl along with its corresponding annotation, release 91. The program STAR (v020201)^17^ was used to align the reads to the genome using default settings and two-pass mode. Since STAR utilizes the gene annotation gtf file, it calculated the number of reads aligned to each gene. Stranded counts were selected.

RNA-SeQC (v1.1.8.1)^14^ was utilized to calculate post-alignment QC metrics. To estimate the proportion of reads belonging to rRNA, the rRNA sequences provided with RNA-SeQC were utilized. RNA-SeQC selects a random subset of the reads and aligns them to the rRNA sequences to estimate the proportion of reads that belong to rRNAs. To similarly calculate a proportion of reads belonging to hgbRNAs, sequences from 12 hgbRNA genes (Supplementary Table S1) from Ensembl (r91) were provided to RNA-SeQC in place of the rRNA sequences. Unlike the rRNAs, the hemoglobin genes are well-annotated; however, a proportion calculated based purely on uniquely-mapped gene counts was not comparable to the rRNA estimates because the gene counts do not include the multi-mapped reads which are present in the hemoglobin genes due to paralogous sequence. The hgbRNA rates estimated using RNA-SeQC can be compared to the rRNA rates since they were calculated the same way relative to total trimmed reads. The proportion of gene counts belonging to hemoglobin transcripts was also calculated (Supplementary Table S2), but a similar proportion cannot be calculated for the rRNAs due to a lack of adequate annotation.

Bioinformatic removal of hgbRNA reads was performed after alignment and gene count calculation by removing 12 hgbRNA genes (Supplementary Table S1) from the read count analysis set prior to normalization, CPM calculations, and edgeR differential expression analyses.

### Data transformation and exploratory analysis

Exploratory data analysis was primarily performed on log_2_-transformed CPM values. We used the CPM function available in the edgeR R package that adds a small count scaled proportional to the library size to avoid taking the log of zero^26^. Multidimensional scaling (MDS) and scatter plots were used to visualize measurement similarities among libraries using log_2_(CPM)^15^. The coefficients of variation (CV) were computed for each library using raw counts. The Wilcoxon signed rank-test was used to compare the medians of CV values.

For analyses utilizing biotype, biotype classifications were retrieved from Ensembl (r91) (https://uswest.ensembl.org/info/genome/genebuild/biotypes.html). The biotypes were binned into a reduced number of more general categories for Fig. 2 (Supplementary Table S3). The only analysis that required normalization accounting for gene length was the gene biotype analysis for RNA copy distribution since it compares transcripts of varying lengths to each other. Gene lengths were derived from the gtf annotation file using GTF2LengthGC.R (https://github.com/dpryan79/Answers/blob/master/SEQanswers_42420/GTF2LengthGC.R) and were used to calculate Transcripts Per Million (TPM) values.

### Gene detection for sensitivity measurement

Uniquely-mapped gene counts from STAR were imported into the R statistical computing environment and normalized by the weighted trimmed mean of M-values (TMM)^27^. For evaluating impact of high (900 ng) or low (250 ng) RNA inputs, we filtered for detected genes using a threshold of >1 CPM in all Control donor libraries with high or low input in Experiment 1 and ≤ 1 CPM in at least one of the alternative input group.

RNA-Seq data from 6 Globin-Zero libraries and 6 RZG libraries from Experiment 1 libraries were selected for a pairwise comparison of library effect on detection with each donor sample. In the paired analysis of Experiment 1, genes were filtered using a threshold of >1 CPM with at least one of library methods. In Experiment 2, we filtered for detected genes using a threshold of >1 CPM in all three technical replicates of at least one sample.

### Differential expression (DE) analysis

For DE analysis, we used uniquely-mapped gene read counts from STAR imported into R and normalized by the weighted trimmed mean of M-values (TMM)^27^. For analysis of Experiment 1 we kept a gene if its uniquely mapped count was larger than 1 CPM in at least one library; this threshold corresponds to about 15 to 25 raw counts. The negative binomial regression model with quasi-likelihood test implemented in the edgeR^19^ R package was employed to find significantly DE genes for the multi-factor analyses. A gene was declared as significantly DE when the associated false discovery (FDR) adjusted p-value was <0.05 and FC > 1.5. In the model for Experiment 1, we included the interaction effect of library preparation methods by diagnosis as well as lane assignment as a blocking factor in addition to all main effects (library prep, input amount, diagnosis). The interaction effect was included to determine which additional genes would be identified by the Globin-Zero library method in the patient group. The blocking factor (lane effects) was included to account for potential lane and other batch biases.

DE analysis of Experiment 2 libraries was performed independently to assess overlap of DEG with the two library methods. Library replicates for C1 and C2 were filtered and analyzed separately for each library method group. A gene was kept and analyzed if it was measured at CPM > 1 in at least one out of the six libraries. EdgeR negative binomial regression models were fitted separately to 6 libraries in a same method group. That is, DE was tested comparing C1 vs C2 using technical replicates within each library method.

## Declarations

### Ethics approval and consent to participate

All protocols were approved by the Institutional Review Board of Oregon Health & Science University. Informed consent was received from all participants in the study.

## Supplementary information

Supplementary Figures.

Supplementary Table S1

Supplementary Table S2

Supplementary Table S3

Supplementary Table S4

Supplementary Table S5

Supplementary Table S6

## Data Availability

Raw sequencing datasets generated during the current study are available through the Sequence Read Archive (SRA) under accession ID PRJNA599020.
