# Supplementary material for: RNA-Seq of human whole blood: Evaluation of globin RNA depletion on Ribo-Zero library method
Source: Sci Rep. 2020 Apr 14;10:6271. doi: 10.1038/s41598-020-62801-6 (PMC7156519; doi:10.1038/s41598-020-62801-6)

Supplementary Information

RNA-Seq of human whole blood: Evaluation of globin RNA depletion on Ribo-Zero library method

Christina A. Harrington[1,2§], Suzanne S. Fei[3], Jessica Minnier[1,4], Lucia Carbone[2,3,5,6], Robert Searles[1,6], Brett Davis[3,5], Kimberly Ogle[7], Stephen R. Planck[7,8], James T. Rosenbaum[7,8,9], and Dongseok Choi[4,7,8,10]

[1]Integrated Genomics Laboratory, Oregon Health & Science University, Portland, Oregon, USA

[2]Department of Molecular & Medical Genetics, Oregon Health & Science University, Portland, Oregon, USA

[3]Bioinformatics & Biostatistics Core, Oregon National Primate Research Center, Oregon Health & Science University, Beaverton, Oregon, USA

[4]OHSU-PSU School of Public Health, Oregon Health & Science University, Portland, Oregon, USA

[5]Knight Cardiovascular Institute, Oregon Health & Science University, Portland, Oregon, USA

[6]Department of Informatics and Medical Epidemiology, Oregon Health & Science University, Portland, Oregon, USA

[7]Casey Eye Institute, Oregon Health & Science University, Portland, Oregon, USA

[8]Department of Medicine, Oregon Health & Science University, Portland, Oregon, USA

[9]Legacy Devers Eye Institute, Legacy Health System, Portland, Oregon, USA

[10]Graduate School of Dentistry, Kyung Hee University, Seoul, Korea

§Corresponding author

Supplementary Figure S1. Library preparation and sequencing design. Technical replication and sequencing design for RNA-seq Experiment 1 and Experiment 2 are shown. A factorial design was used for sequencing lane assignment. **(A)** A ½-fractional factorial design (2^{3-1}) was used to balance samples (6), diagnosis, library RNA input, and library method for Experiment 1. Each colored block corresponds to a lane on the HiSeq2500 platform. Samples designated with asterisks were included for sample number consistency within the lane, but were not included in the data set used for analysis. **(B)** A 2x2 factorial design for Experiment 2 balanced library method and samples C1 and C2.

1A. Experiment 1 design

9 Lanes:

Subdesign 1

D1 (P1 x C1)

	Input	*Library method*	*Diagnosis*
1	250ng	Ribo-Zero Globin	P
2	900ng	Ribo-Zero Globin	C
3	250ng	Ribo-Zero Gold	C
4	900ng	Ribo-Zero Gold	P

Subdesign 2

D1 (P2 x C1)

	Input	*Library method*	*Diagnosis*
1	250ng	Ribo-Zero Globin	C
2	900ng	Ribo-Zero Globin	P
3	250ng	Ribo-Zero Gold	P
4	900ng	Ribo-Zero Gold	C

Subdesign 1

D1 (P3 x C1)

	Input	*Library method*	*Diagnosis*
1	250ng	Ribo-Zero Globin	P
2	900ng	Ribo-Zero Globin	C
3	250ng	Ribo-Zero Gold	C
4*	900ng	Ribo-Zero	X

Subdesign 2

D3 (P1 x C2)

	Input	*Library method*	*Diagnosis*
1	250ng	Ribo-Zero Globin	C
2	900ng	Ribo-Zero Globin	P
3	250ng	Ribo-Zero Gold	P
4	900ng	Ribo-Zero Gold	C

Subdesign 1

D3 (P2 x C2)

	Input	*Library method*	*Diagnosis*
1	250ng	Ribo-Zero Globin	P
2	900ng	Ribo-Zero Globin	C
3	250ng	Ribo-Zero Gold	C
4*	900ng	Ribo-Zero	X

Subdesign 2

D3 (P3 x C2)

	Input	*Library method*	*Diagnosis*
1	250ng	Ribo-Zero Globin	C
2	900ng	Ribo-Zero Globin	P
3	250ng	Ribo-Zero Gold	P
4	900ng	Ribo-Zero Gold	C

Subdesign 1

D5 (P1 x C3)

	Input	*Library method*	*Diagnosis*
1	250ng	Ribo-Zero Globin	P
2	900ng	Ribo-Zero Globin	C
3	250ng	Ribo-Zero Gold	C
4	900ng	Ribo-Zero Gold	P

Subdesign 2

D5 (P2 x C3)

	Input	*Library method*	*Diagnosis*
1	250ng	Ribo-Zero Globin	C
2	900ng	Ribo-Zero Globin	P
3	250ng	Ribo-Zero Gold	P
4	900ng	Ribo-Zero Gold	C

Subdesign 1

D5 (P3 x C3)

	Input	*Library method*	*Diagnosis*
1	250ng	Ribo-Zero Globin	P
2	900ng	Ribo-Zero Globin	C
3	250ng	Ribo-Zero Gold	C
4*	900ng	Ribo-Zero	X

* Not included in analysis

1B. Experiment 2 design

3 Lanes:

	Library method	*Sample*
1	Ribo-Zero Globin	C1
2	Ribo-Zero Globin	C2
3	Ribo-Zero Gold	C1
4	Ribo-Zero Gold	C2

	Library method	*Sample*
1	Ribo-Zero Globin	C1
2	Ribo-Zero Globin	C2
3	Ribo-Zero Gold	C1
4	Ribo-Zero Gold	C2

	Library method	*Sample*
1	Ribo-Zero Globin	C1
2	Ribo-Zero Globin	C2
3	Ribo-Zero Gold	C1
4	Ribo-Zero Gold	C2

Supplementary Figure S2. Multidimensional scaling of Experiment 1 libraries. Multidimensional scaling (MDS) was used to visualize similarity among samples and technical library replicates. **(A)** MDS plot of all 33 libraries pre-pared and sequenced in Experiment 1. **(B)** MDS plot of Experiment 1 libraries after removal of two C1 libraries that failed quality control.

D: Ribo-Zero Gold (RZG); G: Globin-Zero; Color: sample.

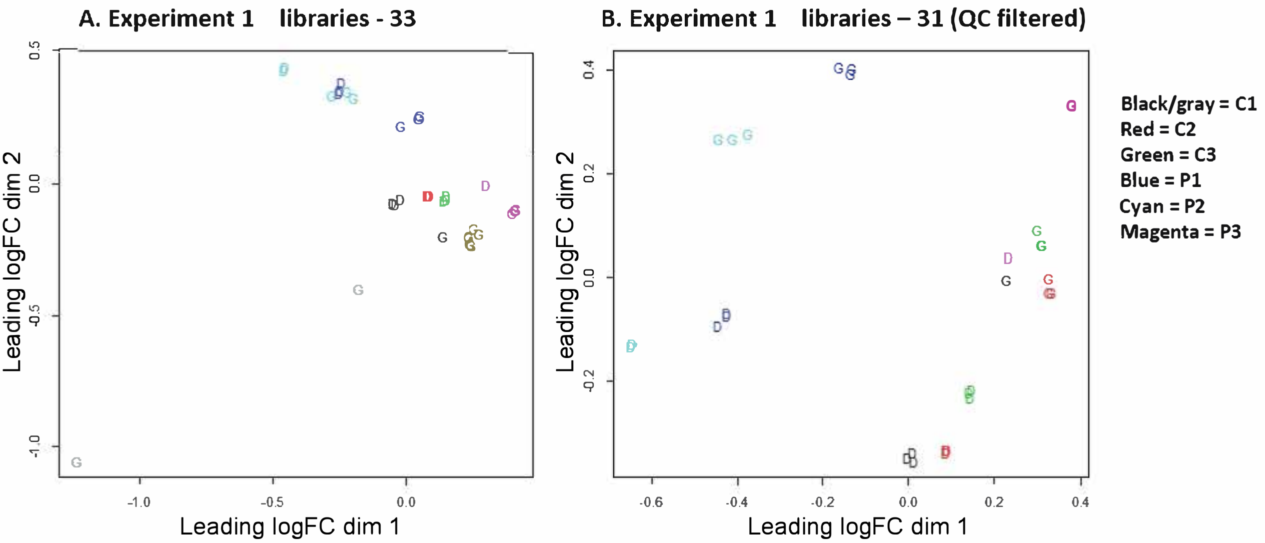

Supplementary Figure S3: Transcript abundance of genes detected in only GZ or RZG libraries in Experiment 2

Reproducible gene detection with library method was determined in Experiment 2 libraries using a filter of CPM > 1 in all 3 method replicates for each sample (C1 and C2). Detection was measured in the presence and absence of hemoglobin (hgb) gene counts. Genes detected with only one method in this experiment were binned according to abundance level: (high: mean (logcpm in RZG) ≥ 3; medium: -1 ≤ mean(logcpm in RZG) < 3; and low: mean(logcpm in RZG) < -1).

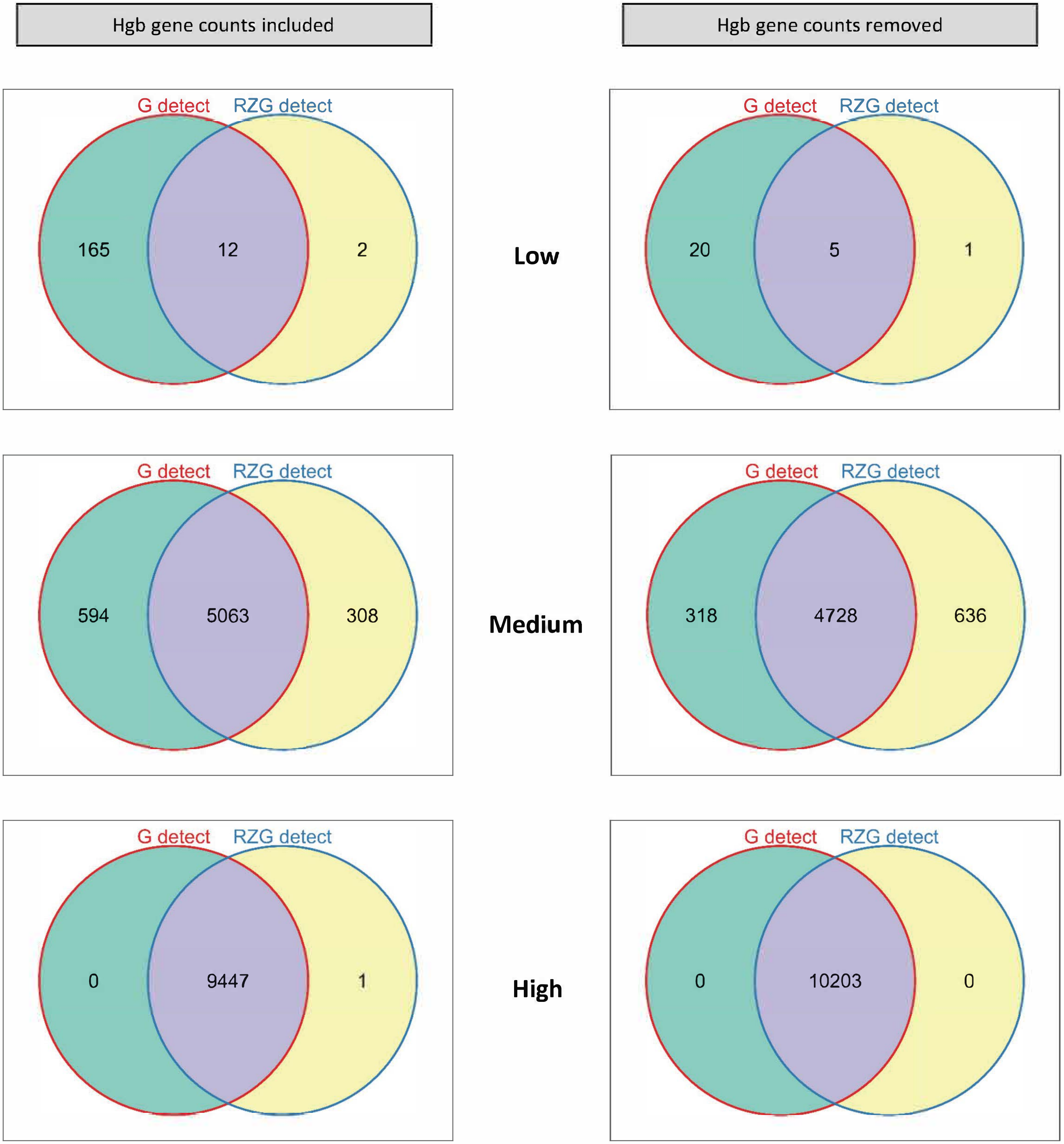

Supplementary Figure S4. Library method has only small effect on differentially expressed genes in Experiment 2. Differential expression analysis of Experiment 2 libraries was performed independently by library method to assess overlap of genes differentially measured between C1 and C2. DE was tested comparing C1 vs C2 using technical replicates within each library method group for a total of 6 library samples per analysis. Samples from each library method group were filtered and analyzed separately. A gene was kept and analyzed if it was measured at CPM > 1 in at least one out of the six samples processed by the same library method. **(a)** DEG for C1 versus C2 with and without the removal of hemoglobin gene counts. A gene was included as significantly differentially expressed when the associated false discovery adjusted p-value (FDR) was < 0.05 and FC > 1.5. GZ = Globin Zero libraries; RZG = RiboZero Gold libraries. **(b)** Biotype distributions for significant DEG with only one library method. Distributions are shown for DE analysis following bioinformatic removal of hemoglobin gene counts in all 12 data sets. **(c)** Biotype distribution for genes passing detection filter (CPM>1) in at least one of six libraries with only one library method and significantly differentially expressed between C1 and C2 with that libarary method.

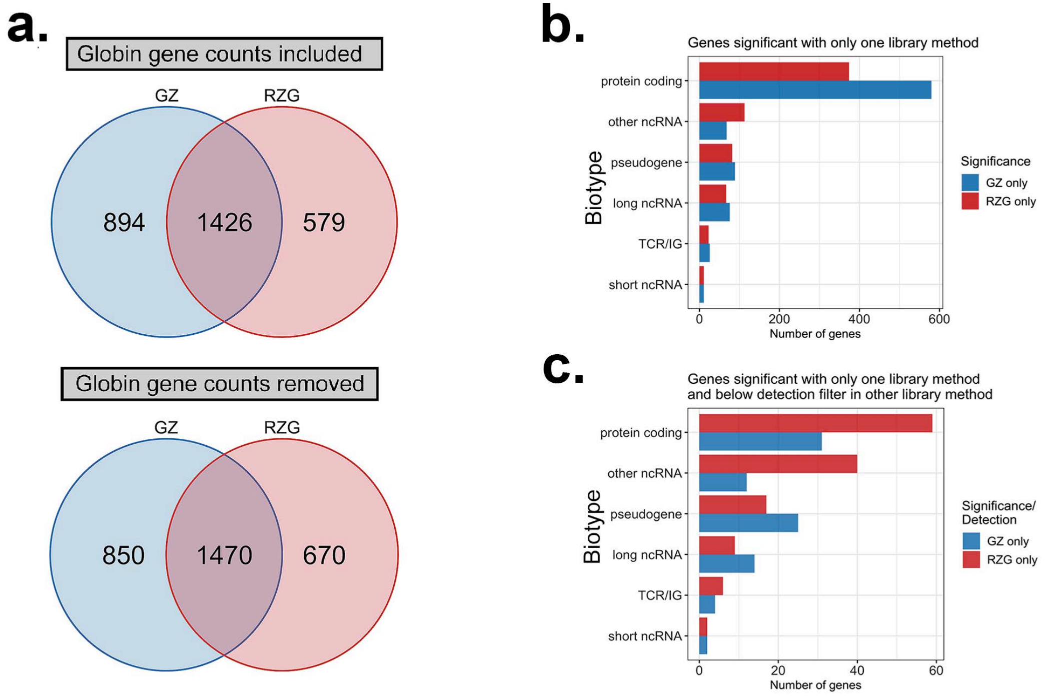

Supplement: Supplementary file 1 — Supplementary Figures. [file 41598_2020_62801_MOESM1_ESM.pdf]
