# Supplementary material for: RNA-Seq of human whole blood: Evaluation of globin RNA depletion on Ribo-Zero library method
Source: Sci Rep. 2020 Apr 14;10:6271. doi: 10.1038/s41598-020-62801-6 (PMC7156519; doi:10.1038/s41598-020-62801-6)
Supplement: Supplementary file 2 — Supplementary Table S1 [file 41598_2020_62801_MOESM2_ESM.docx]

**Supplementary Tables**

**RNA-Seq of human whole blood: Evaluation of globin RNA depletion on Ribo-Zero library method**

Christina A. Harrington^1,2§^, Suzanne S. Fei^3^, Jessica Minnier^4^, Lucia Carbone^2,3,5^, Robert Searles^1^, Brett Davis^3,5^, Kimberly Ogle^6^, Stephen R. Planck^6,7^, James T. Rosenbaum^6,7,8^, and Dongseok Choi^,4,6,7,9^

^1^Integrated Genomics Laboratory, Oregon Health & Science University, Portland, Oregon, USA

^2^Molecular & Medical Genetics, Oregon Health & Science University, Portland, Oregon, USA

^3^Bioinformatics & Biostatistics Core, Oregon National Primate Research Center, Oregon Health & Science University, Beaverton, Oregon, USA

^4^OHSU-PSU School of Public Health, Oregon Health & Science University, Portland, Oregon, USA

^5^Medicine, Knight Cardiovascular Institute, Oregon Health & Science University Portland, Oregon, USA

^6^Casey Eye Institute, Oregon Health & Science University, Portland, Oregon, USA

^7^Department of Medicine, Oregon Health & Science University, Portland, Oregon, USA

^8^Legacy Devers Eye Institute, Legacy Health System, Portland, Oregon, USA

^9^Graduate School of Dentistry, Kyung Hee University, Seoul, Korea

§Corresponding author

**Supplementary Table 1.**

**Human hemoglobin gene family**

| **Ensemble ID** | **Gene name** | **Gene type** | **Gene description** |
| --- | --- | --- | --- |
| ENSG00000206172 | HBA1 | protein_coding | hemoglobin subunit alpha 1 [Source:HGNC Symbol;Acc:HGNC:4823] |
| ENSG00000188536 | HBA2 | protein_coding | hemoglobin subunit alpha 2 [Source:HGNC Symbol;Acc:HGNC:4824] |
| ENSG00000244734 | HBB | protein_coding | hemoglobin subunit beta [Source:HGNC Symbol;Acc:HGNC:4827] |
| ENSG00000229988 | HBBP1 | transcribed_unprocessed_pseudogene | hemoglobin subunit beta pseudogene 1 [Source:HGNC Symbol;Acc:HGNC:4828] |
| ENSG00000223609 | HBD | protein_coding | hemoglobin subunit delta [Source:HGNC Symbol;Acc:HGNC:4829] |
| ENSG00000213931 | HBE1 | protein_coding | hemoglobin subunit epsilon 1 [Source:HGNC Symbol;Acc:HGNC:4830] |
| ENSG00000213934 | HBG1 | protein_coding | hemoglobin subunit gamma 1 [Source:HGNC Symbol;Acc:HGNC:4831] |
| ENSG00000196565 | HBG2 | protein_coding | hemoglobin subunit gamma 2 [Source:HGNC Symbol;Acc:HGNC:4832] |
| ENSG00000206177 | HBM | protein_coding | hemoglobin subunit mu [Source:HGNC Symbol;Acc:HGNC:4826] |
| ENSG00000086506 | HBQ1 | protein_coding | hemoglobin subunit theta 1 [Source:HGNC Symbol;Acc:HGNC:4833] |
| ENSG00000130656 | HBZ | protein_coding | hemoglobin subunit zeta [Source:HGNC Symbol;Acc:HGNC:4835] |
| ENSG00000206178 | HBZP1 | unprocessed_pseudogene | hemoglobin subunit zeta pseudogene 1 [Source:HGNC Symbol;Acc:HGNC:4836] |
